# Co-occurrence of Cardiometabolic Disease Risk Factors: Unhealthy Eating, Tobacco, Alcohol, Sedentary Lifestyle and Socioeconomic Aspects

**DOI:** 10.5935/abc.20190213

**Published:** 2019-10

**Authors:** Maury-Sintjago Eduard, Parra-Flores Julio, Rodríguez-Fernández Alejandra

**Affiliations:** 1 Departamento de Nutrição e Saúde Pública, FACSA, Universidad del Bío-Bío, Chillán - Chile

**Keywords:** Cardiovascular Diseases, Metabolic Syndrome, Morbidity and Mortality, Cluster Analysis, Risk Taking, Alcoholism/epidemiology, Tobacco Use Disorder/epidemiology, Lifestyle, Fast Foods, Sedentarism

Cardiometabolic disease (CMD) is the principal cause of morbi-mortality worldwide. The risk factors in the development of CMD are diverse; understanding them significantly contributes to the design of clinical and/or community strategies for their prevention and/or treatment.^1^ An unhealthy diet, cigarette smoking, sedentary lifestyle, and higher alcohol consumption significantly increase the risk of CMD, cancer, loss of healthy life years, and premature mortality.^2^

In a systematic review by Meader et al.,^3^ 37 studies were proposed in order to evaluate risk behaviors such as smoking, physical inactivity, and unhealthy diet by a meta-analysis. A greater association was found when groups of risk factors co-existed (≥ 4) compared with individual risk factors. Alcohol abuse and smoking were the most commonly identified risk factors. They also reported that the socioeconomic level is a predictor of multiple risks.^3^ On the other hand, in a cohort study of more than 20 years, mortality associated with 1, 2, 3, or 4 risk factors was 1.85 (C I 95%, 1.28-2.68), 2.23 (CI 95%, 1.55-3.20), 2.76 (CI 95%, 1.91-3.99), and 3.49 (CI 95%, 2.31-5.26), respectively. The risk of mortality by CMD was higher than for other causes of death such as cancer.^4^

Among the socioeconomic aspects, the socio-spatial pattern of the points of sale of goods and services is the factor that most predicts unhealthy lifestyles. Macdonald et al.,^5^ in their study in Scotland, show that the distribution of alcohol, fast food, tobacco, and gambling outlets is concentrated in geographical areas with greater socioeconomic deprivation.^5^ Another study shows that in developing countries, the poorest areas have a higher prevalence of inadequate diet and smoking habits and the co-occurrence of risk factors is 20%.^6^

Zwolonsky et al.,^7^ in a study of UK men, found that 72% exhibited combinations of risk factors; physical inactivity (72.8%) together with the lack of fruit and vegetable consumption (73%) was the most common combination. In addition, 29.5% consumed more alcohol than the recommended limit and 25% were smokers.^7^

In this context, Zancheta et al.^8^ detected a strong correlation between alcohol intake and smoking. In addition, unhealthy eating and physical inactivity were the most frequent risk factors. Approximately 3% did not exhibit any risk factors, while 38.0%, 32.9%, 9.4%, and 1.8% showed two to five factors, respectively. It was noteworthy that the highest incidence of these risk factors occurred in girls, older adolescents, those not living with both parents, children of less educated mothers, students attending public schools, and residents of cities in more developed urban areas of the country.^8^

In this issue of the Journal of *Arquivos Brasileiros de Cardiologia*, Stolses et al.^9^ evaluated the co-occurrence of smoking and unhealthy eating in adults in a population sample, showing a high prevalence of inadequate eating (68.4-72.6%) and smoking habits (7.8-15.65%). The occurrence of both risk factors was mainly in men (47%), those residing in the southern part of the country (44.1%), subjects aged 18-39 years (59.4%) and those who consumed alcohol (18.5%). Finally, it is shown that residing in the south (PRadj 1.50; 95% CI 1.18-1.89), not having private health insurance (PRadj 1.14; 95% CI 1.03-1.25), having an abusive consumption of alcohol (PRadj 3.22; 95% CI 2.70-3.85), and reporting a poor state of health (PRadj 1.7; 95% CI 1.18-2.44) were associated significantly with smoking and inadequate diet in Brazilian adults.

In view of the reported scientific evidence, it is increasingly necessary to conduct studies about the co-occurrence of cardiometabolic risk factors because they respond to the multi-causal complexity of the principal health problems worldwide. Therefore, the strategy approach for prevention and/or treatment requires addressing risk factors in multiple rather than individual ways.
